# Supplementary material for: Spatial and temporal evolution of ecotourism development level and its driving factors under the perspective of sustainable development: the case of Ili river valley
Source: PLoS One. 2025 Mar 3;20(3):e0315600. doi: 10.1371/journal.pone.0315600 (PMC11875385; doi:10.1371/journal.pone.0315600)
Supplement: Attachment — Submitted filename: Response to Reviewers.docx [file pone.0315600.s002.docx]

Response to Reviewers

Dear Reviewers:

Thank you for your letter and the comments given by the reviewers on our paper titled “**Spatial and Temporal Evolution of Ecotourism Development Level and Its Driving Factors under the Perspective of Sustainable Development: The Case of Ili River Valley**” (ID: PONE-D-24-13106). These comments are very valuable, are very helpful for us to revise and improve the paper, and also have important guiding significance for our research. We have carefully studied these comments and made revisions, hoping to be recognized.

**Responds to the reviewers' comments:**

***(1)Introduction Section: The specific challenges faced by eco-tourism in the Ili River Valley and the importance of its development should be described in greater detail. This not only includes the pressures of environmental protection and sustainable development but also addresses how to reasonably develop tourism resources while preserving natural ecosystems, thereby achieving a win-win situation for both ecological and economic benefits. Supplementing these aspects will help deepen readers' understanding of the research background and motivation, while also providing a more solid theoretical foundation for subsequent research.***

Thank you for your valuable comments on the introductory section of our manuscript. We are grateful for your suggestions and have taken the following steps to address them: We have expanded the Introduction section to describe in more detail the specific challenges facing ecotourism in the Ili River Valley and the importance of its sustainable development. We believe that these additions will greatly enhance the reader's understanding of the context and motivation for the study. By providing a firmer theoretical foundation, the revised introduction will better support subsequent research in the manuscript. **Relevant changes are reflected on page 4, lines 60-75 of the revised manuscript.** Thank you again for your interest in our work. We look forward to your further review.

***(2) Results Section: The figures and tables could be added or improved to enhance their visual appeal and effectiveness in conveying information. Specifically, adjusting the color scheme could make the different parts of the figures more distinct, thereby improving the readability of the information and the overall aesthetic quality of the figures. Additionally, for figures involving geographic information, considering adjustments to the map projection coordinate systems could more accurately reflect the geographical characteristics of the study area, making the map presentations more consistent with actual conditions.***

Thank you for your valuable suggestions on the results section. We have taken the following steps to address them:

①We have carefully adjusted the color scheme of the charts to make the different sections more distinct. To do this, we have chosen colors with better contrast and visual appeal. This was done to improve the readability of the information in the charts and to improve the overall aesthetics of the charts. In addition, we reassessed and adjusted the map projection coordinate system for charts with geographic information.

②We reviewed the layout and formatting of tables to ensure that they were legible. This included adjusting column widths and font sizes and adding appropriate headings and footnotes where necessary.

We believe that these improvements will greatly enhance the visual appeal of the figures and tables and the effectiveness of the information conveyed. We look forward to your review of the revised manuscript. Thank you again for your attention and guidance.

***(3) References Section: Some of the references cited are from earlier years; it is necessary to reduce the number of older references and increase the inclusion of literature published within the last five years.***

Thank you for your valuable feedback on the references in our manuscripts. We appreciate your concern in this regard and understand the importance of having a balanced and up-to-date list of references.

We have conducted a thorough review of the references and have taken steps to address your concerns. In the revised version, we have carefully evaluated each reference and have tried to reduce the number of older references. We removed references that were less relevant or could be replaced with newer works. We also actively searched for and included additional literature published within the last five years. These new references enhance the currency and relevance of our research and indicate that our work references the latest research in the field. **This change is primarily reflected in documents 1, 2, 7, 8, 11, 21, 33, 34, 35, and 49 in the list of references and has been revised in the text on pages 3, 45-50, page 5, 80-90, and page 10, line 171.**

We look forward to your further review and consideration of our revised manuscript. Thank you again for your feedback and guidance.

***(4) Grammar and Sentence Structure: There are sloppy grammatical and editorial issues everywhere in this paper. Many of them could cause difficulties to the readers.***

Thank you for bringing to our attention the issues regarding grammar and sentence structure in our paper. We sincerely apologize for these shortcomings and understand the potential difficulties they may cause for readers.

We have thoroughly reviewed the manuscript and made significant efforts to correct the sloppy grammatical and editorial issues. We have carefully proofread each sentence, checking for proper grammar, punctuation, and word usage. We have also paid close attention to the overall flow and coherence of the text to ensure that it is presented in a clear and understandable manner.

We are committed to improving the quality of our work and appreciate your feedback, which has been invaluable in helping us to identify and address these areas for improvement. We believe that the revised manuscript now meets a higher standard of grammar and sentence structure and will be more accessible to readers.

Thank you again for your time and consideration. We look forward to your further review of the revised manuscript.
